# What Works to Keep Students in School? A Meta-Analysis of Interventions to Reduce School Absenteeism

**DOI:** 10.3390/bs16050697

**Published:** 2026-05-02

**Authors:** Dan Li, Subhash Singh, Navya Jeldi, Sophie Chang, Mingze Zhu, Kylar Dailey, Aizhan Karabukayeva, Changjie Cai

**Affiliations:** 1 Department of Health Promotion Sciences, Hudson College of Public Health, The University of Oklahoma Health Campus, Oklahoma City, OK 73104, USA; navya-jeldi@ou.edu; 2 Department of Biostatistics & Epidemiology, Hudson College of Public Health, The University of Oklahoma Health Campus, Oklahoma City, OK 73104, USA; subhash-singh@ou.edu; 3 School of Biological Sciences, Dodge Family College of Arts and Sciences, The University of Oklahoma, Norman, OK 73019, USA; sophie.chang-1@ou.edu; 4 Department of Occupational & Environmental Health, Hudson College of Public Health, The University of Oklahoma Health Campus, Oklahoma City, OK 73104, USA; mingze-zhu@ou.edu (M.Z.); changjie-cai@ou.edu (C.C.); 5 Honors College, The University of Oklahoma, Norman, OK 73019, USA; kylar.l.dailey-1@ou.edu; 6 Department of Health Administration & Policy, Hudson College of Public Health, The University of Oklahoma Health Campus, Oklahoma City, OK 73104, USA; aizhan-karabukayeva@ou.edu

**Keywords:** school absenteeism, meta-analysis, randomized controlled trial (RCT), quasi-experimental design (QED), health promotion

## Abstract

(1) Background: Regular school attendance is foundational to students’ academic achievement, social-emotional development, and long-term career success. Yet chronic absenteeism continues to affect a substantial proportion of PreK-12 students in the United States, underscoring the urgent need for evidence-based interventions. (2) Methods: This meta-analysis synthesized findings from 13 randomized controlled trials and quasi-experimental studies published between January 2020 and March 2025 and identified through six digital databases. All included studies evaluated interventions that reported school absenteeism as an outcome. Effect sizes were calculated using Hedges’ *g* and estimated with a random-effects model. To explain variability in effects, meta-regression analyses examined 10 moderators across the following four domains, guided by the ecological systems theory: study-level features (methodological rigor), individual-level characteristics, intervention-level characteristics, and contextual-level characteristics. (3) Results: The overall effect size was 0.091 (95% CI: 0.012–0.170), indicating a small but statistically significant reduction in absenteeism favoring intervention groups. Substantial heterogeneity was observed (*I*^2^ = 73%). Study setting emerged as the only significant moderator, with stronger effects in single-school implementations. Although other moderators were not statistically significant, variation in effect magnitudes suggests meaningful contextual and implementation differences. (4) Conclusions: These interventions produce modest but statistically reliable reductions in absenteeism, with implementation context significantly influencing effectiveness.

## 1. Introduction

Regular school attendance is fundamental to student success, yet an estimated 22% of K-12 students in the United States (approximately 10.8 million students) experienced chronic absenteeism during the 2024–2025 school year (Diliberti et al., 2025). Chronic absenteeism, typically defined as missing at least 10% of school days, has remained substantially elevated compared to pre-pandemic rates of approximately 15% (Malkus, 2024). Missing even two days per month over a nine-month school year results in nearly an entire month of lost in-school time (Gottfried, 2014), creating significant barriers to academic progress and educational engagement. Beyond academic outcomes, chronic absenteeism is associated with weakened socioemotional development (Lindholdt et al., 2024; Santibañez & Guarino, 2021), including reduced educational engagement (Piscitello et al., 2022), lower self-regulation, increased feelings of isolation and disengagement, and diminished interpersonal skills (Kearney et al., 2023; Rahman et al., 2023). As schools increasingly recognize absenteeism as a key indicator of student risk, understanding which interventions effectively reduce chronic absence has become an urgent priority for educators, administrators, and policymakers.

### 1.1. The Problem of Definition and Early Identification

Research on chronic absenteeism depends on both how absenteeism is defined and how interventions are evaluated. The standard definition (i.e., missing at least 10% or more of school days) is commonly used in randomized controlled trials (RCTs) and quasi-experimental studies, yet growing evidence suggests this threshold may be too high to support early identification. Longitudinal analyses show that lower thresholds (3–7%) more effectively identify emerging risk, with the most informative type of absence varying by developmental stage (Wu et al., 2026). These measurement issues have important implications for study design. In RCTs, delayed identification may limit detectable intervention effects by enrolling students whose attendance problems are already entrenched. In quasi-experimental designs (QEDs), variation in thresholds and attendance measures may introduce selection bias and reduce comparability across settings. Accordingly, examining evidence from both RCTs and QEDs allows for a more complete assessment of absenteeism interventions under differing identification rules and real-world constraints.

### 1.2. Questions of Equity and Differential Effectiveness

The effectiveness of attendance interventions must be interpreted within the broader, multilevel context in which absenteeism emerges. Absenteeism arises from a complex interplay of individual, family, school, and community factors (Rapin et al., 1999). Structural barriers, such as poverty, housing instability, transportation challenges, and limited access to healthcare, constrain families’ ability to maintain consistent attendance (Edwards, 2023; Sosu et al., 2021). Therefore, chronic absenteeism affects students unequally across racial, ethnic, and socioeconomic lines. The magnitude of racial and ethnic disparities in chronic absenteeism documented by Ostling (2025) underscores the importance of examining intervention effects by subgroup (Ostling, 2025). With nearly one-third of African American students and over one-quarter of Hispanic students classified as chronically absent, aggregate treatment effects may mask meaningful heterogeneity. Without explicit attention to baseline risk and structural context, intervention evaluations risk overstating effectiveness for groups with fewer barriers while underestimating the supports required for students facing entrenched inequities. Given these disparities in both attendance rates and underlying causes, it matters critically whether existing attendance interventions work equally well for all students.

### 1.3. Intensity and Duration of Interventions

The intensity and duration of interventions also varies substantially, from brief, triggered responses to single instances of chronic absence (Domitrovich et al., 2008) to sustained, multi-year comprehensive programs requiring significant resource investment (Smolkowski et al., 2017). From a practical standpoint, brief interventions are more scalable and cost-effective if they produce effects comparable to longer, more intensive efforts. A phone call home or a single meeting with a family might be sufficient for some students, particularly if absence is situation-specific rather than reflecting deeper underlying barriers. For other students, sustained support over months or years may be necessary to address complex, intersecting challenges. Understanding dose–response relationships can inform resource allocation decisions and help schools balance intervention effectiveness with implementation feasibility. If brief interventions prove nearly as effective as sustained programs, this would support investment in lower-intensity, higher-reach approaches. Conversely, if sustained interventions produce meaningfully better outcomes, this justifies the additional resource commitment they require.

### 1.4. Intervention Context and the Problem of Scale

Research on implementation science has documented that interventions demonstrating efficacy in carefully controlled studies often fail to replicate those effects when implemented at scale (Nilsen et al., 2025). The translation from research to practice involves navigating complex organizational systems, adapting to local contexts, and maintaining fidelity despite varying levels of resource availability and stakeholder commitment (Pearce et al., 2024). These implementation challenges may be particularly salient for attendance interventions, which range from targeted efforts in individual schools to district-wide or statewide initiatives. Single-school implementations may benefit from tighter coordination, stronger relationships between interventionists and school staff, and better cultural fit between the intervention and the specific school community (Guldager et al., 2018). Variability in local capacity means that some schools may lack the personnel, expertise, or resources to implement with adequate quality (Domitrovich et al., 2008). Previous meta-analyses have not systematically examined whether intervention effects differ by implementation setting or scale. This gap limits our ability to advise policymakers about the feasibility of scaling up promising practices or to identify implementation conditions that support stronger effects.

### 1.5. Geographic and Community Context

Schools operate within geographic and community contexts that shape both the nature of attendance challenges and the resources available to address them. Urban schools often contend with concentrated poverty, transportation challenges in large metropolitan areas, safety concerns affecting the journey to and from school, and high student mobility (Gottfried, 2019). Suburban schools typically serve more socioeconomically advantaged populations but may face different challenges related to student stress, pressure, and mental health (Luthar & Latendresse, 2005). Absences in these contexts may relate more to anxiety, school refusal, or mental health challenges than to structural barriers like transportation or housing instability. Small cities and rural communities face distinct attendance challenges shaped by geographic isolation, limited access to health and mental health services, transportation barriers, and school climates that influence students’ sense of belonging. Findings from rural districts suggest that absenteeism is not driven by a single factor, but rather by the interaction of family circumstances, health needs, school culture, and uneven implementation of attendance supports, with these constraints becoming more pronounced at the secondary level (Gleaves et al., 2025).

Regional variation adds another layer of complexity. School funding levels, policy environments, and cultural norms around education differ substantially across U.S. regions (Favero & Kagalwala, 2025). These regional differences in resources, policy context, and educational culture may shape both attendance patterns and intervention effectiveness. An intervention that works well in a well-resourced suburban district in the Northeast may require substantial adaptation to function effectively in an under-resourced rural district in the South. Yet these geographic and regional differences have not been systematically examined as moderators of intervention effectiveness, leaving practitioners uncertain about how to adapt evidence-based practices to their specific geographic contexts.

### 1.6. The State of Evidence on Attendance Interventions

Schools have implemented various approaches to improve attendance, ranging from behavioral and school climate supports to academic interventions, health services, family engagement programs, and community partnerships (Eklund et al., 2022). However, until recently, evidence base for these interventions has remained limited. Early systematic reviews identified relatively few rigorous evaluations and concluded that the field lacked sufficient evidence to draw a firm conclusion about effectiveness (Maynard et al., 2013; Sutphen et al., 2010). More recently, Eklund et al. (2022) conducted a meta-analysis of attendance interventions and reported a small but positive overall effect (*g* = 0.25). Importantly, this analysis revealed substantial heterogeneity across studies (*I*^2^ = 92.72%), indicating wide variation in intervention effects (Eklund et al., 2022).

Despite this progress, critical questions remain unanswered. First, questions of equity remain largely unexamined. For instance, given that students of color and students experiencing poverty face disproportionately higher rates of chronic absenteeism (U.S. Department of Education, 2025), understanding whether existing interventions work equitably across various populations represents both a methodological and ethical imperative. Second, the field has limited evidence about dose–response relationships—whether longer, more intensive interventions produce meaningfully better outcomes than brief, targeted approaches. Third, we lack systematic understanding of how implementation context influences intervention effectiveness. Do interventions work equally well when scaled from individual schools to district-wide implementations? Finally, geographic and community context may matter substantially, yet these factors have not been systematically and adequately examined across studies.

### 1.7. The Current Study

This meta-analysis extends previous work by systematically examining factors that may explain heterogeneity in attendance intervention effects. Building on Eklund et al.’s (2022) foundation while addressing several of their identified limitations, we focus exclusively on studies employing rigorous research designs to provide more confident estimates of causal effects while reducing heterogeneity related to methodological variation.

Drawing on ecological systems theory, which conceptualizes individual development as shaped by a set of interconnected environmental systems (Bronfenbrenner, 2000), we view student attendance as influenced by interacting factors at the individual level and across broader contextual levels. Accordingly, we organized moderators into four theoretically informed domains: study-level features (research design, publication type), individual-level characteristics (grade range, racial/ethnic composition, socioeconomic status), intervention-level characteristics (intervention type, dose intensity), and contextual-level characteristics (study setting, urbanicity, geographic region).

Study-level features were examined as methodological characteristics rather than as components of the ecological model itself, consistent with our approach in prior related studies (Lemberger-Truelove et al., 2026; Lemberger-Truelove et al., 2025). Individual-level characteristics reflect student-level differences that may shape absenteeism risk and responsiveness to intervention. Intervention-level characteristics represent differences in the mechanisms and intensity through which programs may affect attendance. Contextual-level characteristics capture school- and community-level conditions that may influence attendance patterns and implementation.

Accordingly, our specific research questions are:

What is the overall effectiveness of school-based interventions on student attendance outcomes in studies employing randomized controlled trials (RCTs) and quasi-experimental designs (QEDs)?

Do intervention effects differ by research design quality (RCT vs. QED) or publication type (peer-reviewed articles vs. dissertations)?

Do intervention effects differ by grade range (elementary, middle, high school, or mixed), racial/ethnic composition (general/mixed vs. predominantly students of color), or socioeconomic status (high vs. lower economic disadvantage)?

Do intervention effects differ by intervention type (behavioral/school climate, academic/career readiness, or health/clinical) or dose intensity (brief/low dose, long duration, or unclear)?

Do intervention effects differ by study setting (single school, multi-school, multi-district/statewide, or mixed/unclear), urbanicity (urban, suburban, small city, or mixed/unclear), or geographic region (South, multi-region, West, Midwest, unknown, and Northeast)?

By examining these questions systematically, we aim to provide evidence-based guidance about the conditions under which attendance interventions are most effective. The findings have direct implications for practitioners selecting interventions tailored to their specific contexts and student needs, policymakers allocating limited resources to the most promising approaches, and researchers designing future studies that can fill identified gaps in the knowledge base.

## 2. Materials and Methods

### 2.1. Literature Search Strategy

We worked with the university librarian to ensure a thorough search. Because our research spans interdisciplinary fields, we included six digital databases across education, health, interdisciplinary studies, intervention-focused research, and dissertations and theses: ERIC, PsycINFO, PubMed, Scopus, ClinicalTrials.gov, and ProQuest.

The search strategy combined three concept blocks using Boolean operators: (1) school attendance problems, (2) interventions or prevention efforts, and (3) rigorous quantitative study designs. Within each block, controlled vocabulary and keyword terms were combined using OR, and the three blocks were combined using AND. Truncation and proximity operators were applied where supported by the database. Because search syntax varied across databases due to differences in search functionality, six database-specific search strings were developed rather than a single uniform string. The full search strings are available from the authors upon request.

### 2.2. Eligibility Criteria

Following the database search, studies were screened against predefined inclusion and exclusion criteria. The inclusion criteria were as follows: (a) employed rigorous quantitative designs (e.g., RCTs, including both individual- and cluster-level trials; QEDs, such as difference-in-differences, propensity score matching, interrupted time series, natural experiments, or pretest–posttest designs with comparison groups); (b) focused on early childhood or PreK-12 children and adolescents; (c) evaluated an intervention, program, treatment, prevention effort, practice, or strategy; (d) included a comparison condition (e.g., control group, waitlist, or practice-as-usual); (e) reported school attendance-related outcomes; and (f) were conducted in the United States, published in English, and published between January 2020 and March 2025, when the research team concluded the search. This search window was selected to focus the review on the most recent evidence base and contemporary intervention contexts related to school absenteeism, particularly during a period characterized by substantial changes in schooling, student needs, and attendance challenges during and following the COVID-19 pandemic.

The exclusion criteria were as follows: non-experimental studies, meta-analyses, studies conducted outside the United States, non-English publications, studies published before January 2020, and studies not reporting attendance-related outcomes. This study was retrospectively registered on the Open Science Framework (OSF).

### 2.3. Article Screening and Data Extraction

**Figure 1:**
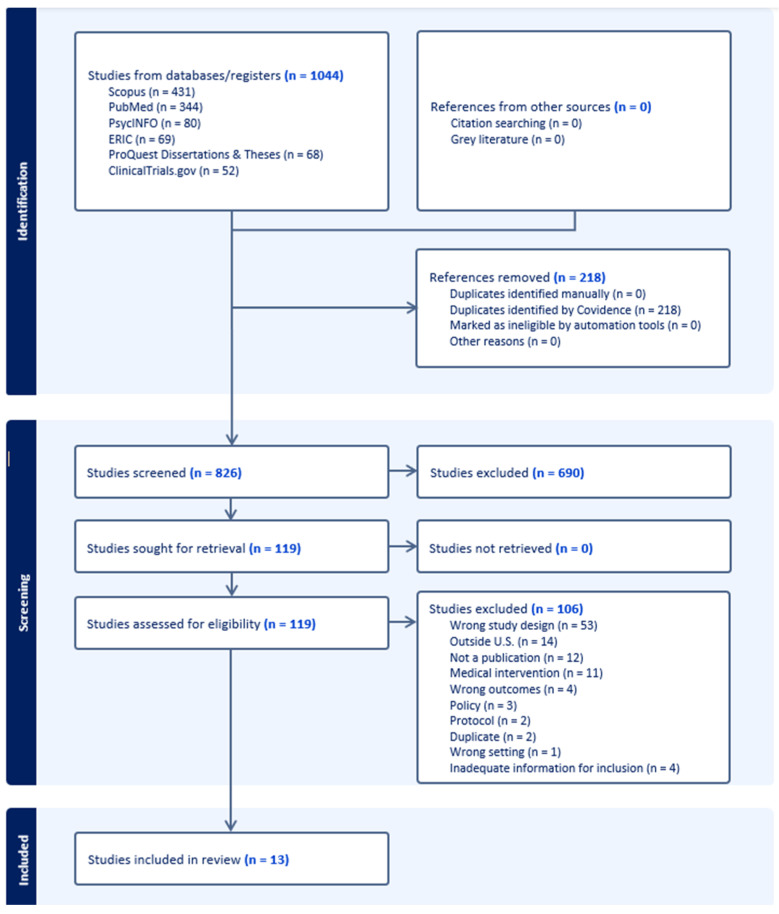
PRISMA flow diagram. The flow diagram summarizes the identification, screening, eligibility assessment, and inclusion of studies in the meta-analysis conducted between January 2020 and March 2025.

Covidence (2025), a web-based collaboration software platform that streamlines the production of systematic and other literature reviews, was used to organize and screen the literature. A total of 1044 studies were imported from six digital databases for screening (see Figure 1 for the PRISMA flow diagram with additional details). Covidence automatically identified and removed 218 duplicate records. 

Screening proceeded in three stages: (a) title and abstract screening, (b) full-text review, and (c) final determination of study inclusion. A screening team was formed consisting of the first through sixth authors. Each article was screened by two team members (selected from the third, fourth, fifth, and sixth authors). Any discrepancies were discussed during full team meetings until consensus was reached regarding inclusion. Following the full-text review, the first and second authors reviewed all included studies to verify alignment with the design criteria and to ensure that sufficient statistical information was reported or could be computed for subsequent data extraction and coding. Final decisions regarding inclusion for meta-analysis were made at this stage. Given that we included two study designs, RCT and QED, the risk of bias was assessed separately. The Cochrane RoB 2 tool was used to evaluate RCTs, and ROBINS-I was used to assess QED studies.

As we screened the articles, we developed a codebook and revised it iteratively across multiple rounds. Once the coding categories were finalized, the last author used Python 3.7 to extract them from all included articles. To ensure accuracy, each content-based extracted code was independently verified by two team members. The first author independently verified all statistical codes. The Python script is available from the authors upon request.

### 2.4. Outcome Measures

Outcomes were primarily reported as school attendance days or rates, or school absence days or rates. During data analysis, the direction of effect sizes was reversed when necessary to ensure consistent interpretation of outcomes across studies. Across studies, data were primarily drawn from administrative and school-based records. In addition to administrative data, some studies incorporated teacher-reported measures, parent-reported data, and student self-reported surveys.

### 2.5. Data Analysis

The first author used Comprehensive Meta-Analysis (Version 4) (Borenstein, 2022) to conduct the data analyses. The standardized mean difference (Hedges’ *g*) was used as the effect size index. A random-effects model was employed because the included studies were assumed to represent a random sample from a broader population of potential studies, and the goal was to generalize findings to that population (Borenstein et al., 2021; Hedges & Vevea, 1998). Following the overall analysis, we conducted a series of meta-regression analyses to examine potential moderator effects.

To reduce potential heterogeneity arising from differences in study design, and to account for the practical challenges of implementing RCTs in clustered school settings, we included only RCTs and QEDs that incorporated both an intervention group and a comparison (control) group. Because pre–post correlation coefficients were not consistently reported across studies, we assumed a correlation of 0.5 for all included studies. Missing data were addressed at the study level. When required statistical information was not reported, we calculated effect sizes from the available statistics whenever possible. If sufficient information was not available to compute an effect size, the study was excluded from the quantitative synthesis.

### 2.6. Publication Bias and Selective Reporting

To reduce potential publication bias and to better reflect the full scope of available evidence, we intentionally included both peer-reviewed journal articles and dissertations or theses in our literature search. Accordingly, the final sample included a mix of peer-reviewed journal articles (*n* = 10) and student dissertations (*n* = 3).

**Figure 2:**
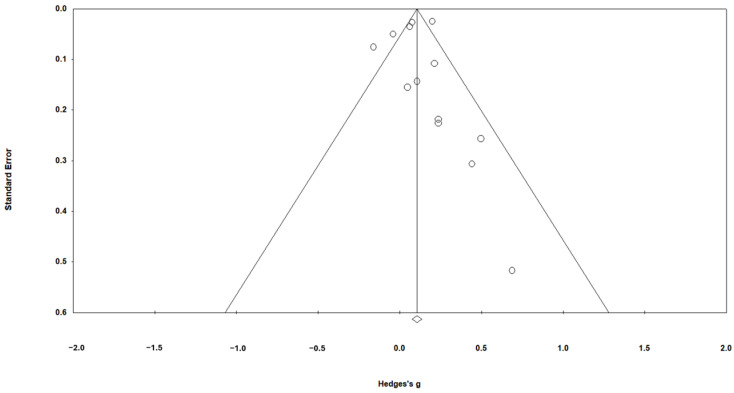
Funnel plot of standard error by Hedges’ *g*. Note. Each open circle represents an individual study’s effect size (Hedges’ *g*) plotted against its standard error. The vertical line indicates the overall pooled effect size estimated from the random-effects model. The diagonal lines represent the 95% confidence limits around the summary effect, forming the expected “funnel” shape in the absence of publication bias. The diamond at the bottom represents the overall mean effect size.

First, we used a funnel plot of standard error by Hedges’ *g* (see Figure 2) to assess potential publication bias. Visual inspection suggested some asymmetry, with a relative scarcity of smaller studies reporting negative or null effects and a greater concentration of small studies showing positive effects. However, interpretation of funnel plot asymmetry should be made with caution. The number of included studies was relatively small (*n* = 13), and funnel plots have limited power to reliably detect publication bias when fewer than 10–20 studies are available. In addition, observed asymmetry may reflect true heterogeneity, small-study effects, or methodological differences rather than publication bias per se.

Next, we calculated Rosenthal’s Classic Fail-safe N to assess the robustness of the overall effect. The observed studies (*n* = 13) yielded a statistically significant combined effect (*Z* = 5.40, *p* < 0.001). The fail-safe N test indicated that 86 additional null-effect studies would be required to raise the overall *p*-value above the alpha level of 0.05. These findings provide only tentative evidence of robustness and should be interpreted cautiously given the small number of included studies.

## 3. Results

### 3.1. Overall Findings

**Figure 3:**
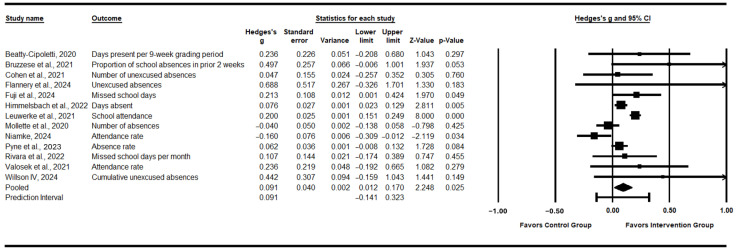
Forest plot of effect sizes for the included studies (Beatty-Cipoletti, 2020; Bruzzese et al., 2021; Cohen et al., 2021; Flannery et al., 2024; Fuji et al., 2024; Himmelsbach et al., 2022; Leuwerke et al., 2021; Mollette et al., 2020; Niamke, 2024; Pyne et al., 2023; Rivara et al., 2022; Valosek et al., 2021; Willson IV, 2024). Note. Each square represents an individual study’s effect size (Hedges’ *g*), and the horizontal line through each square indicates its 95% confidence interval. The size of each square reflects the study’s weight in the meta-analysis. The diamond at the bottom represents the overall pooled effect size estimated from the random-effects model, with its width indicating the 95% confidence interval. Positive effect sizes favor the intervention group.

The analysis is based on 13 studies (see Figure 3). The mean effect size was 0.091 (95% CI: 0.012–0.170), favoring the intervention group. Because the confidence interval did not include 0, the effect was statistically significant (*Z* = 2.248, *p* = 0.025). There was significant heterogeneity among studies, *Q*(12) = 44.388, *p* < 0.001. The *I*^2^ statistic was 73%, indicating that approximately 73% of the observed variance reflects real differences in effect sizes rather than sampling error. The estimated between-study variance (*τ*^2^) was 0.009, corresponding to a standard deviation of true effect sizes (*τ*) of 0.097 in Hedges’ *g* units.

### 3.2. Moderator Analyses

Because substantial heterogeneity was observed, we conducted a series of meta-regressions to explore potential subgroup differences based on 10 selected moderators. These moderators were grouped into four domains: study-level features (research design, publication type), individual-level characteristics (grade range, race/ethnicity, socioeconomic status [SES]), intervention-level characteristics (intervention type, dose intensity), and contextual-level characteristics (study setting, urbanicity, U.S. region). Study setting was the only moderator that reached statistical significance. However, these findings should be interpreted cautiously. With only 13 studies meeting the inclusion criteria, substantial between-study heterogeneity, and largely non-significant moderator tests, the moderator analyses are best understood as exploratory and hypothesis-generating rather than confirmatory. Accordingly, non-significant moderator effects should not be interpreted as evidence that contextual factors are unimportant, but rather as reflecting the limited statistical power available to detect potentially meaningful between-study differences. Although most moderator effects were not statistically significant, variation in the magnitude of effect sizes may still offer useful descriptive signals for future research.

#### 3.2.1. Study-Level Features

In this study, we included only randomized controlled trials (RCTs, *n* = 6), including both individual-level and cluster-level randomization, and quasi-experimental designs (QEDs, *n* = 7) to reduce heterogeneity that could arise from substantially different research designs. Research design was not a significant moderator of intervention effects, *Q*(1) = 1.48, *p* = 0.2242. Mixed-effects subgroup analyses indicated that the pooled effect size for RCTs (*g* = 0.174, 95% CI [0.021, 0.328]) was larger than that for QEDs (*g* = 0.055, 95% CI [−0.061, 0.171]); however, the between-group difference was not statistically significant.

To reduce publication bias, we included both peer-reviewed journal articles (*n* = 10) and doctoral dissertations (*n* = 3). Publication type was not a significant moderator of intervention effects, *Q*(1) = 1.79, *p* = 0.1804. Subgroup analyses indicated that the pooled effect size for peer-reviewed journal articles (*g* = 0.109, 95% CI [0.029, 0.190]) was larger than that for dissertations (*g* = −0.035, 95% CI [−0.230, 0.160]); however, the between-group difference was not statistically significant.

#### 3.2.2. Individual-Level Characteristics

Grade range was not a significant moderator of intervention effects, *Q*(3) = 0.11, *p* = 0.9905. Grade range was coded into four categories: elementary (PreK–5, *n* = 1), middle school (6–8, *n* = 2), high school (9–12, *n* = 4), and mixed or unclear (*n* = 6) grade ranges. Although pooled effects were slightly larger for middle school *g* = 0.125 (95% CI [−0.146, 0.395]) and mixed or unclear grade ranges *g* = 0.117 (95% CI [−0.031, 0.264]) than for elementary *g* = 0.076 (95% CI [−0.216, 0.368]) and high school *g* = 0.084 (95% CI [−0.157, 0.326]), confidence intervals overlapped substantially, and none of the differences were statistically significant.

Race/ethnicity was not a statistically significant moderator of intervention effects, *Q*(1) = 3.34, *p* = 0.0677, although the result approached significance. Race/ethnicity was coded into two categories: general or mixed samples with no race-defined focus (*n* = 9), and samples predominantly composed of students of color (*n* = 4). The pooled effect size for general or mixed samples was *g* = 0.132 (95% CI [0.047, 0.216]), whereas the pooled effect size for predominantly students-of-color samples was *g* = −0.006 (95% CI [−0.127, 0.115]). Although the effect size was numerically larger for general or mixed samples, the between-group difference was not statistically significant.

Socioeconomic status (SES) was not a statistically significant moderator of intervention effects, *Q*(2) = 2.56, *p* = 0.2779. SES was coded into three categories: high economically disadvantaged (*n* = 5), lower economically disadvantaged (*n* = 1), and unknown or unclear SES (*n* = 7). The pooled effect size for samples with high economic disadvantage was *g* = 0.044 (95% CI [−0.081, 0.169]), whereas the pooled effect size for samples with lower economic disadvantage was *g* = 0.688 (95% CI [−0.353, 1.728]); notably, this subgroup was represented by only one study. The pooled effect size for samples with unknown or unclear SES was *g* = 0.153 (95% CI [0.015, 0.291]). Although the effect size was numerically largest for samples with lower economic disadvantage, the between-group difference was not statistically significant, and the estimate for this subgroup should be interpreted with caution given the limited number of studies.

#### 3.2.3. Intervention-Level Characteristics

Intervention type was not a statistically significant moderator of intervention effects, *Q*(2) = 1.81, *p* = 0.4043. Intervention type was coded into three categories: behavior and school climate supports (*n* = 4), academic and career readiness (*n* = 7), and health and clinical care (*n* = 2). The largest pooled effect size was observed for health and clinical care interventions (*g* = 0.217, 95% CI [−0.066, 0.500]), although this effect was not statistically significant. Academic and career readiness interventions demonstrated a smaller but statistically significant pooled effect (*g* = 0.098, 95% CI [0.011, 0.185]). In contrast, behavior and school climate support interventions showed a near-zero and non-significant pooled effect (*g* = −0.006, 95% CI [−0.194, 0.182]).

Dose intensity was not a statistically significant moderator of intervention effects, *Q*(2) = 5.29, *p* = 0.0711, although the result approached statistical significance. Dose intensity was categorized as brief or low dose (triggered or limited-contact approaches, or any intervention with a stated duration of 6 months or less; *n* = 8), long duration (the stated duration is more than 6 months; *n* = 4), and unclear duration (frequency is given but duration is not stated or is ambiguous; *n* = 1). The largest pooled effect size was observed for long-duration interventions (*g* = 0.126, 95% CI [0.011, 0.242]), which demonstrated a statistically significant positive effect. Brief or low-dose interventions also showed a statistically significant pooled effect (*g* = 0.110, 95% CI [0.006, 0.214]). In contrast, interventions with unclear duration yielded a negative and non-significant pooled effect (*g* = −0.160, 95% CI [−0.385, 0.064]).

#### 3.2.4. Contextual-Level Characteristics

Study setting was a statistically significant moderator of intervention effects, *Q*(3) = 8.38, *p* = 0.0388. Study setting was coded into four categories: single school (*n* = 3), multi-school (single district/metro/area; *n* = 4), multi-district/statewide (*n* = 3), and mixed/non-school or unclear settings (*n* = 3). The largest pooled effect size was observed for studies conducted in single-school settings (*g* = 0.351, 95% CI [0.006, 0.696]). Studies conducted in mixed/non-school or unclear settings also demonstrated the second-largest positive pooled effect (*g* = 0.213, 95% CI [0.024, 0.401]). A smaller but statistically significant effect was observed for studies conducted at the multi-district/statewide level (*g* = 0.129, 95% CI [0.022, 0.236]). In contrast, studies implemented in multi-school settings showed a near-zero and non-significant pooled effect (*g* = −0.016, 95% CI [−0.119, 0.087]). Overall, these findings suggest that intervention effects varied meaningfully by study setting, with stronger effects observed in more localized or less formally structured contexts. However, subgroup estimates should be interpreted with caution due to limited statistical power. This model explained approximately 37% of the between-study variance in effect sizes.

Urbanicity was not a statistically significant moderator of intervention effects, *Q*(3) = 5.78, *p* = 0.1228. Urbanicity was coded into four categories: urban (*n* = 4), suburban (*n* = 3), small city (*n* = 1), and mixed or unclear settings (*n* = 5). The largest pooled effect size was observed for studies conducted in small cities (*g* = 0.688, 95% CI [−0.335, 1.710]); however, this estimate was based on one study and associated with a wide confidence interval. Studies conducted in mixed or unclear settings also demonstrated a positive and statistically significant pooled effect (*g* = 0.186, 95% CI [0.070, 0.302]). In contrast, studies conducted in urban settings showed a small and non-significant pooled effect (*g* = 0.043, 95% CI [−0.046, 0.132]), as did studies conducted in suburban settings (*g* = 0.021, 95% CI [−0.134, 0.175]).

U.S. region was not a statistically significant moderator of intervention effects, *Q*(5) = 1.55, *p* = 0.9072. Studies were categorized into six regions: South (*n* = 2), Multi-region (*n* = 2), West (*n* = 4), Midwest (*n* = 3), Unknown (*n* = 1), and Northeast (*n* = 1). The largest pooled effect size was observed for studies conducted in the Northeast (*g* = 0.442, 95% CI [−0.247, 1.130]), although this estimate was based on one study and associated with substantial uncertainty. Studies conducted across multiple regions also demonstrated a positive pooled effect (*g* = 0.209, 95% CI [−0.154, 0.572]). Smaller, non-significant pooled effects were observed for studies conducted in the West (*g* = 0.139, 95% CI [−0.098, 0.375]), Midwest (*g* = 0.084, 95% CI [−0.127, 0.296]), and Unknown regions (*g* = 0.076, 95% CI [−0.265, 0.416]). The smallest pooled effect was observed for studies conducted in the South (*g* = 0.038, 95% CI [−0.258, 0.335]).

### 3.3. Risk of Bias (RoB) Assessment

Using the Cochrane RoB 2 domains (ratings include Low Risk, Some Concerns, and High Risk), the RCTs generally demonstrated adequate randomization procedures, with several studies clearly describing computer-generated or blocked allocation; however, incomplete reporting of sequence generation or allocation concealment in some trials resulted in judgments of “some concerns,” and one study was rated “high risk” due to departures from true random assignment. Deviations from intended interventions were mostly rated as “some concerns,” most often because blinding was not feasible and adherence or contamination was insufficiently detailed. Risk related to missing outcome data was generally “low” where follow-up rates were high and administrative data were used, though some studies raised “some concerns” due to attrition or limited reporting. Outcome measurement was most often judged “low risk” when based on school records, whereas reliance on parent- or self-reported absences introduced “some concerns.” Selective reporting was frequently rated as “some concerns” when trial registration or pre-specified protocols were not clearly documented, with “low risk” observed in prospectively registered trials. Overall, the body of RCT evidence reflected predominantly “some concerns,” with one study judged to be at “high risk” of bias.

Using the ROBINS-I domains (ratings include Low, Moderate, Serious, Critical, and Unclear), the quasi-experimental studies clearly classified intervention and comparison groups, resulting in a “low” risk of bias for intervention classification; however, the lack of randomization introduced important threats to internal validity, particularly confounding and selection bias, which were commonly rated “moderate” to “serious” despite the use of matching or longitudinal analytic strategies in some studies to strengthen baseline comparability. Outcome measurement was generally judged to be “low” risk, with occasional concerns about measurement quality, whereas risk related to missing data ranged from “low” to “serious.” Reporting of deviations from intended interventions and selective reporting was often insufficiently described, leading to “unclear” judgments in these domains. Overall, the body of evidence reflected predominantly “moderate” to “serious” risk of bias.

## 4. Discussion

While the mean effect size was small, it was statistically significant, indicating an overall reduction in school absenteeism across the included studies. However, given the substantial heterogeneity, this pooled effect is best understood as an average across a diverse body of studies rather than as evidence of a consistent intervention effect. Notably, this pattern was observed across studies with varying objectives, including those that directly targeted school attendance and those that assessed absenteeism as one of several outcomes.

For instance, among the included studies, some targeted academic and career readiness, including an action civics intervention (Cohen et al., 2021), a financial education and coaching program for low-income, single-mother households (Fuji et al., 2024), sending teacher-written postcards home (Himmelsbach et al., 2022), a college access program (Leuwerke et al., 2021), an accelerated middle school intervention (Mollette et al., 2020), a summer learning program (Pyne et al., 2023), and a competitive robotics club (Willson IV, 2024).

Some targeted behavior and school climate supports, including a bullying intervention for improving grades and attendance (Beatty-Cipoletti, 2020), a Check-In/Check-Out (CICO) intervention for improving high school student outcomes (Flannery et al., 2024), a meditation intervention for improving psychological stress and academic achievement (Valosek et al., 2021), and planned mentorship for improving academic achievement and behavior (Niamke, 2024).

Others targeted health and clinical care, including a web-based asthma intervention (Bruzzese et al., 2021) and collaborative care for persistent postconcussive symptoms and their impact on academic functioning (Rivara et al., 2022).

These interventions align with the Whole School, Whole Community, Whole Child (WSCC) model (Centers for Disease Control and Prevention, 2024), which positions schools as important contexts for students’ academic, behavioral, and physical health development. In this review, the WSCC model served as a conceptual framework for organizing intervention targets, but not as evidence that interventions within a given domain operate through a common mechanism or produce uniform effects. Taken together, the findings are consistent with the view that absenteeism may be influenced through a range of school-based and health-related strategies, even when attendance is not the primary focus of intervention.

### 4.1. Integrating Findings with Prior Research

#### 4.1.1. Interpreting the Overall Intervention Effect

Given that this meta-analysis includes only 13 studies, its findings should be regarded as exploratory in light of the limited evidence base. Therefore, caution is warranted when interpreting these results and when considering their generalizability to the broader population.

The pooled effect of *g* = 0.091 is smaller than Eklund et al.’s (2022) estimate of *g* = 0.25. Although this effect is small by conventional benchmarks, this effect warrants careful interpretation. Chronic absenteeism emerges from intersecting individual, family, school, and community factors (Kearney et al., 2023), including structural barriers like poverty, housing instability, and limited healthcare access (Edwards, 2023; Sosu et al., 2021). Expecting large effects from single interventions may be unrealistic given this complexity.

Moreover, small effects translate into meaningful population-level impacts. With 10.8 million students experiencing chronic absenteeism annually (Diliberti et al., 2025), even modest improvements yield substantial cumulative benefits. However, the modest magnitude suggests chronic absenteeism cannot be resolved through isolated strategies. The substantial heterogeneity reinforces this: effectiveness varies considerably by design, population, and context. This points toward coordinated, multi-level approaches addressing various, intersecting barriers.

#### 4.1.2. Methodological Rigor and the Strength of Evidence

Although research design and publication type were not statistically significant moderators, the observed trends suggest meaningful implications for the field. RCTs demonstrated larger pooled effects than QEDs, underscoring the continued value of rigorous experimental designs in producing more stable and internally valid estimates of intervention impact, consistent with Lemberger-Truelove et al.’s (2025) findings. Similarly, while publication type did not significantly influence outcomes, peer-reviewed studies showed slightly stronger effects, highlighting the potential role of methodological scrutiny in strengthening research quality. Together, these findings reinforce the importance of prioritizing rigorous design and peer-reviewed scholarship while maintaining inclusive evidence synthesis practices to ensure both credibility and comprehensiveness in the evidence base.

#### 4.1.3. Individual-Level Variation in Intervention Effects

Grade range did not emerge as a significant moderator, aligning with some prior research (e.g., Lemberger-Truelove et al., 2026; Lemberger-Truelove et al., 2025); however, the magnitude of effects appeared somewhat mixed across grade categories in different studies. Although pooled estimates suggested slightly stronger effects in certain grade ranges, the differences were small and characterized by substantial overlap in confidence intervals. Taken together, these findings indicate that, within the current evidence base, intervention effectiveness does not meaningfully differ by grade level. At the same time, the limited number of studies in several grade categories may have reduced statistical power to detect true subgroup differences, warranting cautious interpretation and further investigation.

One concerning finding relates to race and ethnicity. Although the result approached but did not reach statistical significance, the pattern warrants attention. Interventions showed positive effects in general or mixed samples but null effects in samples predominantly composed of students of color. A similar pattern emerged with respect to SES. However, these patterns are inconsistent with related literature. For instance, Lemberger-Truelove et al. (2025) found that the magnitude of effect sizes for social and emotional interventions delivered by school counselors to improve students’ development was greater in settings with high racial/ethnic representation than in those with low representation, and greater in low-SES settings compared to higher-SES settings.

Critically, these findings challenge a common assumption that evidence-based practices demonstrating effectiveness in one population will generalize to others. Our results suggest they may not. The field needs interventions explicitly designed with and for underserved communities, addressing structural barriers directly rather than treating them as implementation complication. This means not just better interventions but tailored interventions: developed through participatory process, providing material supports alongside behavioral strategies, and addressing school climate as core components rather than add-ons. They should also center culturally responsive practices, so that intervention design and delivery are aligned with students’ identities, lived experiences, and community contexts.

At the same time, this pattern warrants cautious interpretation. The subgroup analysis was based on a limited number of studies, and the observed difference did not reach conventional levels of statistical significance. Moreover, the number of studies conducted in general or mixed samples was more than double the number conducted in samples predominantly composed of students of color. As such, any apparent differences in effects across these groups should be considered exploratory and hypothesis-generating, and may reflect inequities in the evidence base rather than true differences in intervention effectiveness.

#### 4.1.4. Intervention-Level Characteristics and Mechanisms of Impact

Intervention type did not significantly moderate outcomes, although some descriptive patterns emerged. These findings partially align with research identifying disengagement as a key driver (Piscitello et al., 2022). Interventions making school more engaging and relevant, through academic support, career connections, authentic learning, may address disengagement more directly than interventions focused solely on climate or behavior. Students seeing connections between education and future goals, experiencing academic success, and finding learning interesting attend more regularly.

However, weak behavioral/climate effects are puzzling given research linking climate, belonging, and student–adult relationships to attendance (Kearney et al., 2023; Rahman et al., 2023). This may reflect implementation challenges rather than inherent ineffectiveness. Creating genuinely welcoming climates requires sustained whole-school effort: discipline policy changes, culturally responsive training, relationship restructuring, and curricular adaptation. If “climate” interventions consisted of surface-level changes rather than deep reforms, weak effects are unsurprising.

Promising but inconclusive health findings warrant investigation. Health problems (e.g., acute illnesses and chronic conditions like asthma) contribute substantially to absenteeism, particularly in under-resourced communities with limited healthcare (Edwards, 2023). School-based health services and care coordination could theoretically reduce health-related absences, but we identified too few rigorous evaluations. This represents an important gap, particularly since addressing health barriers may be most critical for students showing smallest responses to other intervention types, especially those in high-poverty and under-resourced communities.

Although the WSCC framework is useful for organizing the intervention literature, its analytical value in this review is limited because interventions within the same domain often differ in their components, intensity, implementation, and likely mechanisms of action. Accordingly, the WSCC model is more useful for situating absenteeism within a broader multi-system framework than for identifying which specific intervention mechanisms are most effective. The current evidence suggests that absenteeism may be influenced through multiple domains, but it does not yet support strong conclusions about “what exactly works” or “why.” Future research should consistently and adequately report intervention logic models, core components, and implementation features more precisely to enable mechanism-informed comparison.

Dose intensity approached significance, with both brief and long-duration interventions showing similar positive effects. The absence of a dose–response relationship is notable: longer interventions produced no meaningful advantage over brief ones. A similarly nonsignificant relationship has also been observed in other studies (Lemberger-Truelove et al., 2026; Lemberger-Truelove et al., 2025). This pattern suggests two possibilities. First, intervention quality and targeting may matter more than duration. Brief interventions delivered at the right moment to appropriately identified students may be as effective as sustained programs. Second, optimal dose likely varies by barrier type. Students facing temporary, situational barriers (acute illness, isolated transportation issues) may need only brief support, while those experiencing chronic, intersecting challenges require sustained intervention.

#### 4.1.5. Contextual-Level Influences on Intervention Effects

Study setting emerged as the only statistically significant moderator, explaining approximately 37% of between-study variance. Single-school interventions produced the largest effects, substantially exceeding multi-district/statewide programs, while multi-school implementations showed near-zero effects. These findings empirically confirm what implementation science predicts: interventions lose effectiveness at scale as organizational complexity increases (Nilsen et al., 2025; Pearce et al., 2024). One reason may be that scaling involves extending interventions that succeeded in a limited range of contexts under stringent controls to a broader range of contexts in less restrictive settings (Ryan et al., 2024). As a result, scaling evidence-based practices in education has proven difficult to achieve in practice (Ryan et al., 2024).

Schools may represent relatively self-contained organizational contexts in which implementation fidelity is easier to monitor, including adherence to intervention content, coverage, frequency, and duration, along with moderators such as intervention complexity, facilitation strategies, quality of delivery, and participant responsiveness (Carroll et al., 2007). Durlak and DuPre (2008) further argue that organizational capacity, together with training and technical assistance, is central to effective implementation, and they identify several organizational features linked to implementation, including work climate, organizational norms regarding change, integration of new programming, shared vision, shared decision-making, coordination with other agencies, communication, formulation of tasks, leadership, program champion, and managerial/supervisory/administrative support. This suggests that more bounded settings with greater relational proximity, such as single schools, may be better positioned to coordinate and sustain the organizational conditions that support high-quality implementation.

However, our results reveal a pattern more complex than simple linear attenuation. The particularly weak effects in multi-school, single-district settings, which are weaker even than statewide implementations, might suggest that mid-level scaling may represent the most challenging implementation context. This could reflect coordination burdens without the infrastructure, dedicated resources, or policy support that sometimes accompany state-level initiatives.

The magnitude of difference between single-school and multi-school effects is substantial, approaching the size of the overall pooled effect itself. This pattern suggests that implementation context may be a major determinant of observed effects, not merely a background condition. One plausible interpretation is that interventions delivered in more bounded single-school settings may achieve stronger effects because implementation is easier to coordinate, monitor, and sustain than in multi-school delivery contexts. Conversely, when multiple schools are included in a study, preexisting contextual differences across sites may introduce greater heterogeneity in implementation conditions, which could in turn attenuate implementation quality and observed intervention effects.

### 4.2. Implications

#### 4.2.1. Modest but Meaningful Reductions in Absenteeism

Across the synthesized studies, absenteeism decreases on average, indicating that at-tendance is modifiable and that improvement is achievable through intervention, though effects are typically modest. This modest-but-positive pattern is still policy-relevant because even small reductions in absenteeism can generate meaningful academic and long-term benefits when scaled across schools or districts. At the same time, these implications should be interpreted cautiously. Given the small number of included studies, substantial between-study heterogeneity, and uneven rigor across the evidence base, the present synthesis provides limited leverage for drawing strong conclusions about the magnitude, stability, and generalizability of intervention effects. Future work should therefore move beyond asking “whether interventions work” to identifying “how,” “for whom,” and “under what conditions” they work best, especially given uneven rigor and inconsistent effects across contexts and subgroups (Middleton et al., 2026).

A key implication is the importance of implementation context. Evidence suggesting stronger effects in single-school settings points to potential advantages of localized coordination, stronger implementation fidelity, and better contextual alignment, although this interpretation remains provisional. For practitioners and policymakers, this supports prioritizing context-sensitive implementation strategies (e.g., clear delivery structures, monitoring, and adaptation to local constraints) alongside more rigorous evaluation designs that can explain heterogeneity rather than simply document it (Kearney et al., 2023; Middleton et al., 2026).

#### 4.2.2. Cross-Domain Gains and the Need for Collaboration

The fact that interventions across multiple domains can improve attendance, even when attendance is not the primary target, reinforces the idea that absenteeism is multi-determined and embedded in interacting systems (academic functioning, well-being, health, family processes, school climate, and community conditions). From this perspective, attendance can function as an early signal of stress or misalignment across these systems, which helps explain why a variety of intervention approaches sometimes yield convergent attendance improvements (Kearney et al., 2023). At the same time, the current evidence base is too limited to support strong conclusions about which domains are most effective or which mechanisms are primarily responsible for change. This complexity nevertheless implies that durable attendance gains likely require coordinated, cross-role collaboration among educators, families, student support staff, administrators, and, when relevant, health and community partners (Kearney et al., 2023; Middleton et al., 2026).

For example, in the domain of academic and career readiness, interventions that increase relevance, enrichment, and future-oriented goal alignment may improve attendance by strengthening perceived value and routine participation (D’Agostino et al., 2026). Regarding the behavior and school climate supports, relational and engagement-focused strategies (e.g., mentoring, counseling supports, family engagement) appear promising because they recur across the evidence base, even as effect sizes vary by context, again underscoring the need for locally aligned implementation and cross-stakeholder coordination (Middleton et al., 2026). “Light-touch” behavioral communication strategies can complement these efforts when targeted; for example, parent text messaging reduced chronic absenteeism in preschool settings with stronger impacts for students with lower baseline attendance, suggesting targeted deployment may be particularly effective for high-need groups (Kalil et al., 2021).

In the domain of health and clinical care, health-related barriers are common and actionable, making healthcare–school partnerships an important part of coordinated attendance strategies. Screening for attendance risk in pediatric clinical settings can identify medically related absences and connect families to supports earlier (Goldman et al., 2025), while coordinated school nursing and health supports align naturally with tiered models that emphasize safe participation (McCutcheon, 2026). Clinical interventions can be effective when emotional/behavioral drivers are central (Jakobsen et al., 2025). This reinforces the need for coordinated systems of care and rigorous, consistent attendance measurement to support credible conclusions and effective scaling (Jakobsen et al., 2025; Johnsen et al., 2024).

### 4.3. Limitations and Future Research Directions

A primary limitation of this study is the small evidence base (13 studies), which constrains statistical precision and limits the ability to test moderators that are central to the conclusion, namely, “how,” “for whom,” and “under what conditions” interventions are most effective. Specifically, the search strategy relied exclusively on database searches and did not include forward or backward citation searching, hand-searching, or efforts to identify unpublished studies. As a result, some relevant evidence may have been missed. In addition, the review was restricted to RCTs and QEDs, studies published between 2020 and 2025, and studies conducted in the United States only. These criteria substantially narrowed the evidence base. With few studies, meta-analyses are often underpowered for detecting meaningful subgroup differences and contextual effects (Hedges & Pigott, 2001), and publication-bias assessments are less reliable (Sterne et al., 2011). Therefore, the non-significant moderator findings should be interpreted cautiously and not over-interpreted as evidence of the absence of moderation.

Future research could strengthen the practical and policy relevance of this evidence by expanding the pool of eligible studies in several ways: (a) searching additional digital databases, (b) seeking unpublished studies, (c) including a broader range of research designs that still permit effect-size computation and pooling, (d) extending the search across a longer time period, and (e) broadening the geographic scope to include international studies. These steps align with best-practice guidance for systematic review conduct and transparent reporting (Page et al., 2021) and may help reduce the risk of publication bias by capturing more grey literature and null findings (Hopewell et al., 2007). As the evidence base grows, future syntheses will be better positioned to evaluate implementation context (e.g., single-school vs. multi-site delivery) and other conditions that may explain differential impacts, an emphasis directly implied by the current conclusion.

A second major limitation is the substantial heterogeneity, which reduces the interpretability of a single overall pooled estimate and suggests that intervention effects likely differ by domain, population, and implementation context (Higgins et al., 2003; Higgins & Thompson, 2002). At the same time, this heterogeneity is not only a methodological complication but also a substantive finding. The wide variation in effect sizes indicates that absenteeism interventions do not operate uniformly across studies and that their impacts are likely shaped by differences in intervention targets, implementation processes, and local conditions. Thus, the modest pooled effect is only part of the story. Equally important is the pattern of divergence across studies, which suggests that absenteeism interventions may work differently depending on whom they target, which barriers they address, and the settings in which they are implemented.

Although our moderators were informed by an ecological systems perspective, that framework served primarily to organize the moderators conceptually rather than to imply that studies within the same category shared a common mechanism of change. Indeed, the pooled studies differed substantially in intervention content, sample characteristics, and implementation context. For example, moderator groupings based on grade range necessarily collapsed diverse student populations into broad categories such as elementary, middle, and high school, even though meaningful differences may exist within those groupings. More fine-grained categorization might help explain some of the observed divergence, but this was not feasible given the limited number of available studies.

Accordingly, future research should classify and compare interventions using more precise, theory-informed moderators. As the evidence base grows, future reviews could use prespecified subgroup analyses or meta-regression to test contextual explanations, while interpreting such findings cautiously (Sun et al., 2010; Thompson & Higgins, 2002). In parallel, primary studies should report implementation features more consistently (e.g., fidelity, adaptations, coordination structures), because stronger implementation/process evidence is essential for explaining why effects vary across contexts (Moore et al., 2015; Skivington et al., 2021). This would also help reduce the risk of bias.

## 5. Conclusions

This meta-analysis provides timely, preliminary, practice- and policy-relevant evidence on the effectiveness of interventions that include school attendance or absence as an outcome. Although the overall effects are modest, even small reductions in absenteeism may yield meaningful academic and long-term benefits when implemented at scale. The findings also suggest that implementation context may matter, particularly the advantages observed in single-school settings. One plausible interpretation is that localized coordination, stronger implementation fidelity, and better contextual alignment may enhance impact. At the same time, because this meta-analysis includes only 13 studies, the findings should be considered exploratory given the limited evidence base. In addition, given the substantial heterogeneity across studies, future research should move beyond identifying “whether interventions work” to understanding “how,” “for whom,” and “under what conditions” they are most effective. Accordingly, this review is best understood as hypothesis-generating rather than confirmatory. Its primary contribution is to identify preliminary patterns, highlight key sources of heterogeneity, and clarify priorities for future research. For practitioners and policymakers, the findings support the value of context-sensitive implementation strategies and rigorous evaluation designs to strengthen attendance-related outcomes. Overall, these findings contribute to the growing evidence base on attendance improvement efforts and underscore the need for sustained, contextually responsive approaches to addressing school absenteeism.

## Data Availability

No new data were created or analyzed in this study.

## Abbreviations

The following abbreviations are used in this manuscript:

RCT	Randomized controlled trial
QED	Quasi-experimental design
SES	Socioeconomic status
RoB	Risk of Bias

## References

[B1-behavsci-16-00697] Beatty-Cipoletti R. A. (2020). A quantitative causal comparative study: A bullying intervention on grades and attendance. Doctoral dissertation.

[B2-behavsci-16-00697] Borenstein M. (2022). Comprehensive meta-analysis software. Systematic reviews in health research.

[B3-behavsci-16-00697] Borenstein M., Hedges L. V., Higgins J. P. T., Rothstein H. R. (2021). Introduction to meta-analysis.

[B4-behavsci-16-00697] Bronfenbrenner U. (2000). Ecological systems theory. Encyclopedia of psychology.

[B5-behavsci-16-00697] Bruzzese J.-M., George M., Liu J., Evans D., Naar S., DeRosier M. E., Thomas J. M. (2021). The development and preliminary impact of CAMP air: A web-based asthma intervention to improve asthma among adolescents. Patient Education and Counseling.

[B6-behavsci-16-00697] Carroll C., Patterson M., Wood S., Booth A., Rick J., Balain S. (2007). A conceptual framework for implementation fidelity. Implementation Science: IS.

[B7-behavsci-16-00697] Centers for Disease Control and Prevention (2024). Whole school, whole community, whole child (WSCC).

[B8-behavsci-16-00697] Cohen A. K., Fitzgerald J. C., Ridley-Kerr A., Maker Castro E., Ballard P. J. (2021). Investigating the impact of generation citizen’s action civics education program on student academic engagement. The Clearing House: A Journal of Educational Strategies, Issues and Ideas.

[B9-behavsci-16-00697] Covidence (2025). Covidence systematic review software, Veritas Health Innovation, Melbourne, Australia.

[B10-behavsci-16-00697] D’Agostino E. M., Manyara R., Erickson T., Chen A., Peters A., Blakemore A., Maina E., Vargas C., Zimmerman K. O. (2026). A comprehensive outside-of-school intervention to promote school attendance and achievement during the COVID-19 pandemic. Public Health in Practice.

[B11-behavsci-16-00697] Diliberti M. K., Chu L., Rainey L. R., DiNicola S. E., Lake R. J., Schwartz H. L. (2025). Chronic absenteeism still a struggle in 2024–2025.

[B12-behavsci-16-00697] Domitrovich C. E., Bradshaw C. P., Poduska J. M., Hoagwood K., Buckley J. A., Olin S., Romanelli L. H., Leaf P. J., Greenberg M. T., Ialongo N. S. (2008). Maximizing the implementation quality of evidence-based preventive interventions in schools: A conceptual framework. Advances in School Mental Health Promotion.

[B13-behavsci-16-00697] Durlak J. A., DuPre E. P. (2008). Implementation matters: A review of research on the influence of implementation on program outcomes and the factors affecting implementation. American Journal of Community Psychology.

[B14-behavsci-16-00697] Edwards D. S. (2023). Another one rides the bus: The impact of school transportation on student outcomes in Michigan. Education Finance and Policy.

[B15-behavsci-16-00697] Eklund K., Burns M. K., Oyen K., DeMarchena S., McCollom E. M. (2022). Addressing chronic absenteeism in schools: A meta-analysis of Evidence-Based Interventions. School Psychology Review.

[B16-behavsci-16-00697] Favero N., Kagalwala A. (2025). The politics of school funding: How state political ideology is associated with the allocation of revenue to school districts. Educational Policy.

[B17-behavsci-16-00697] Flannery K. B., Kato M. M., Kittelman A., Sampson N. K., McIntosh K. (2024). Check-In/Check-Out (CICO) in high school: Results from a small randomized controlled trial. Behavioral Disorders.

[B18-behavsci-16-00697] Fuji K. T., White N. D., Packard K. A., Kalkowski J. C., Walters R. W. (2024). Effect of a financial education and coaching program for low-income, single mother households on child health outcomes. Healthcare.

[B19-behavsci-16-00697] Gleaves G., Sanford I., Stewart C. (2025). Student absenteeism in a rural school district. Doctoral dissertation.

[B20-behavsci-16-00697] Goldman V. E., Antoun J., Rezvan P. H., Fang K. (2025). School absenteeism screening in the healthcare setting: Linking education and medicine. Journal of School Health.

[B21-behavsci-16-00697] Gottfried M. A. (2014). Chronic absenteeism and its effects on students’ academic and socioemotional outcomes. Journal of Education for Students Placed at Risk (JESPAR).

[B22-behavsci-16-00697] Gottfried M. A. (2019). Chronic absenteeism in the classroom context: Effects on achievement. Urban Education.

[B23-behavsci-16-00697] Guldager J. D., Andersen P. T., Von Seelen J., Leppin A. (2018). Physical activity school intervention: Context matters. Health Education Research.

[B24-behavsci-16-00697] Hedges L. V., Pigott T. D. (2001). The power of statistical tests in meta-analysis. Psychological Methods.

[B25-behavsci-16-00697] Hedges L. V., Vevea J. L. (1998). Fixed- and random-effects models in meta-analysis. Psychological Methods.

[B26-behavsci-16-00697] Higgins J. P. T., Thompson S. G. (2002). Quantifying heterogeneity in a meta-analysis. Statistics in Medicine.

[B27-behavsci-16-00697] Higgins J. P. T., Thompson S. G., Deeks J. J., Altman D. G. (2003). Measuring inconsistency in meta-analyses. BMJ.

[B28-behavsci-16-00697] Himmelsbach Z., Weisenfeld D., Lee J. R., Hersh D., Sanbonmatsu L., Staiger D. O., Kane T. J. (2022). Your child missed learning the alphabet today: A randomized trial of sending teacher-written postcards home to reduce absences. Journal of Research on Educational Effectiveness.

[B29-behavsci-16-00697] Hopewell S., McDonald S., Clarke M. J., Egger M. (2007). Grey literature in meta-analyses of randomized trials of health care interventions. The Cochrane Database of Systematic Reviews.

[B30-behavsci-16-00697] Jakobsen S., Tølbøll K. B., Thastum M., Lomholt J. J. (2025). Cognitive behavioral interventions for school attendance problems: A systematic review and meta-analysis.

[B31-behavsci-16-00697] Johnsen D. B., Lomholt J. J., Heyne D., Jensen M. B., Jeppesen P., Silverman W. K., Thastum M. (2024). The effectiveness of modular transdiagnostic cognitive behavioral therapy versus treatment as usual for youths displaying school attendance problems: A randomized controlled trial. Research on Child and Adolescent Psychopathology.

[B32-behavsci-16-00697] Kalil A., Mayer S. E., Gallegos S. (2021). Using behavioral insights to increase attendance at subsidized preschool programs: The show up to grow up intervention. Organizational Behavior and Human Decision Processes, Nudges and Choice Architecture in Organizations.

[B33-behavsci-16-00697] Kearney C. A., Dupont R., Fensken M., Gonzálvez C. (2023). School attendance problems and absenteeism as early warning signals: Review and implications for health-based protocols and school-based practices. Frontiers in Education.

[B34-behavsci-16-00697] Lemberger-Truelove M. E., Li D., Kim H., Hill D. D., Dickson R., Kang Z. (2026). School mental health interventions for adolescents: A meta-analysis of effectiveness and relevant moderators. Adolescents.

[B35-behavsci-16-00697] Lemberger-Truelove M. E., Li D., Kim H., Wills L., Thompson K., Lee Y. (2025). Meta-analysis of social and emotional learning interventions delivered by school counselors. Journal of Counseling & Development.

[B36-behavsci-16-00697] Leuwerke W. C., Ingleby L. D., Tillery C. Y., Cech T. G., Sibaouih C. M. (2021). Narrowing the college readiness gap: Assessing GEAR UP Iowa’s intermediate impact on underserved students. Journal of Education for Students Placed at Risk (JESPAR).

[B37-behavsci-16-00697] Lindholdt L., Svendsen K., Rothausen K. W., Bech B. H. (2024). Social well-being and problematic school absence among Danish adolescents: A nationwide cross-sectional study. Scandinavian Journal of Public Health.

[B38-behavsci-16-00697] Luthar S. S., Latendresse S. J. (2005). Children of the affluent: Challenges to well-being. Current Directions in Psychological Science.

[B39-behavsci-16-00697] Malkus N. (2024). Long COVID for public schools: Chronic absenteeism before and after the pandemic.

[B40-behavsci-16-00697] Maynard B. R., McCrea K. T., Pigott T. D., Kelly M. S. (2013). Indicated truancy interventions for chronic truant students: A campbell systematic review. Research on Social Work Practice.

[B41-behavsci-16-00697] McCutcheon K. (2026). Using a multi-tiered system of support to address chronic absenteeism in students with chronic health conditions. NASN School Nurse.

[B42-behavsci-16-00697] Middleton A., Watson M., Anderson J. K. (2026). What school-based interventions work to improve attendance in secondary school students with persistent absence? A systematic review. Frontiers in Child and Adolescent Psychiatry.

[B43-behavsci-16-00697] Mollette M., Villa B., Cate D. (2020). Accelerated middle school programs: Preliminary indicators of long-term academic benefits for over-age youth. Journal of Education for Students Placed at Risk (JESPAR).

[B44-behavsci-16-00697] Moore G. F., Audrey S., Barker M., Bond L., Bonell C., Hardeman W., Moore L., O’Cathain A., Tinati T., Wight D., Baird J. (2015). Process evaluation of complex interventions: Medical Research Council guidance. BMJ.

[B45-behavsci-16-00697] Niamke M. (2024). Effects of planned mentorship on African-American high school students’ academic achievement and behavior. Ph.D. thesis.

[B46-behavsci-16-00697] Nilsen P., Kirk J. W., Gunnarsson K. U., Thomas K. (2025). Tempering implementation optimism: Distinguishing between efficacy and effectiveness in implementation research. Implementation Science Communications.

[B47-behavsci-16-00697] Ostling F. (2025). Assessing predictors of chronic absenteeism in students of color through quantitative methods. Doctoral dissertation.

[B48-behavsci-16-00697] Page M. J., McKenzie J. E., Bossuyt P. M., Boutron I., Hoffmann T. C., Mulrow C. D., Shamseer L., Tetzlaff J. M., Akl E. A., Brennan S. E., Chou R., Glanville J., Grimshaw J. M., Hróbjartsson A., Lalu M. M., Li T., Loder E. W., Mayo-Wilson E., McDonald S., Moher D. (2021). The PRISMA 2020 statement: An updated guideline for reporting systematic reviews. BMJ.

[B49-behavsci-16-00697] Pearce N., Monks H., Alderman N., Hearn L., Burns S., Runions K., Francis J., Cross D. (2024). ‘It’s all about context’: Building school capacity to implement a whole-school approach to bullying. International Journal of Bullying Prevention.

[B50-behavsci-16-00697] Piscitello J., Kim Y. K., Orooji M., Robison S. (2022). Sociodemographic risk, school engagement, and community characteristics: A mediated approach to understanding high school dropout. Children and Youth Services Review.

[B51-behavsci-16-00697] Pyne J., Messner E., Dee T. S. (2023). The dynamic effects of a summer learning program on behavioral engagement in school. Education Finance and Policy.

[B52-behavsci-16-00697] Rahman M. A., Renzaho A. M. N., Kundu S., Awal M. A., Ashikuzzaman M., Fan L., Ahinkorah B. O., Okyere J., Kamara J. K., Mahumud R. A. (2023). Prevalence and factors associated with chronic school absenteeism among 207,107 in-school adolescents: Findings from cross-sectional studies in 71 low-middle and high-income countries. PLoS ONE.

[B53-behavsci-16-00697] Rapin I., Steinberg M., Waterhouse L. (1999). Consistency in the ratings of behaviors of communicatively impaired autistic and non-autistic preschool children. European Child & Adolescent Psychiatry.

[B54-behavsci-16-00697] Rivara F. P., Marcynyszyn L. A., Wang J., Chrisman S. P. D., Hilt R., Zatzick D. F., Johnson A. M., Jinguji T., Quitiquit C., McCarty C. A. (2022). Effect of collaborative care for persistent postconcussive symptoms on academic function: A randomized clinical trial. Journal of School Health.

[B55-behavsci-16-00697] Ryan A., Prieto-Rodriguez E., Miller A., Gore J. (2024). What can Implementation Science tell us about scaling interventions in school settings? A scoping review. Educational Research Review.

[B56-behavsci-16-00697] Santibañez L., Guarino C. M. (2021). The effects of absenteeism on academic and social-emotional outcomes: Lessons for COVID-19. Educational Researcher.

[B57-behavsci-16-00697] Skivington K., Matthews L., Simpson S. A., Craig P., Baird J., Blazeby J. M., Boyd K. A., Craig N., French D. P., McIntosh E., Petticrew M., Rycroft-Malone J., White M., Moore L. (2021). A new framework for developing and evaluating complex interventions: Update of Medical Research Council guidance. BMJ.

[B58-behavsci-16-00697] Smolkowski K., Seeley J. R., Gau J. M., Dishion T. J., Stormshak E. A., Moore K. J., Falkenstein C. A., Fosco G. M., Garbacz S. A. (2017). Effectiveness evaluation of the Positive Family Support intervention: A three-tiered public health delivery model for middle schools. Journal of School Psychology.

[B59-behavsci-16-00697] Sosu E. M., Dare S., Goodfellow C., Klein M. (2021). Socioeconomic status and school absenteeism: A systematic review and narrative synthesis. Review of Education.

[B60-behavsci-16-00697] Sterne J. A. C., Sutton A. J., Ioannidis J. P. A., Terrin N., Jones D. R., Lau J., Carpenter J., Rucker G., Harbord R. M., Schmid C. H., Tetzlaff J., Deeks J. J., Peters J., Macaskill P., Schwarzer G., Duval S., Altman D. G., Moher D., Higgins J. P. T. (2011). Recommendations for examining and interpreting funnel plot asymmetry in meta-analyses of randomised controlled trials. BMJ.

[B61-behavsci-16-00697] Sun X., Briel M., Walter S. D., Guyatt G. H. (2010). Is a subgroup effect believable? Updating criteria to evaluate the credibility of subgroup analyses. BMJ: British Medical Journal.

[B62-behavsci-16-00697] Sutphen R. D., Ford J. P., Flaherty C. (2010). Truancy interventions: A review of the research literature. Research on Social Work Practice.

[B63-behavsci-16-00697] Thompson S. G., Higgins J. P. T. (2002). How should meta-regression analyses be undertaken and interpreted?. Statistics in Medicine.

[B64-behavsci-16-00697] U.S. Department of Education (2025). Chronic absenteeism: Supporting student attendance and combatting chronic absenteeism in our nation’s schools.

[B65-behavsci-16-00697] Valosek L., Nidich S., Grant J., Peterson M., Nidich R. (2021). Effect of meditation on psychological stress and academic achievement in high school students: A randomized controlled study. Education.

[B66-behavsci-16-00697] Willson IV A. E. (2024). An action research study exploring the academic and social-emotional effects of offering a competitive robotics club in a public middle school. Doctoral dissertation.

[B67-behavsci-16-00697] Wu T., Christina W., Thomas S. (2026). The chronic(les) of absenteeism measurement: Unpacking the many measures of attendance and evidence for a lower chronic absenteeism threshold.

